# Retrospective study of men with *E. coli* UTI treated with an oral antibiotic, and risk for a new prescription or hospital admission due to UTI

**DOI:** 10.1080/02813432.2020.1718301

**Published:** 2020-01-30

**Authors:** Filip Jansåker, Jonas Bredtoft Boel, Niels Frimodt-Møller, Jenny Dahl Knudsen

**Affiliations:** a Department of Clinical Microbiology, Rigshospitalet, University of Copenhagen, Copenhagen, Denmark;; b Department of Clinical Microbiology, Hvidovre Hospital, University of Copenhagen, Hvidovre, Denmark;; c Department of Clinical Microbiology, Herlev Hospital, University of Copenhagen, Herlev, Denmark

Dear Sir,

In light of the increased resistance to fluoroquinolones other oral non-fluoroquinolone antibiotics are increasingly being used for lower urinary tract infection (UTI) in men in Scandinavia [1–4], but without clinical studies supporting the use of these antibiotics for men with UTI. A recent study by Montelin et al. [5] from Sweden compared different non-fluoroquinolone oral antibiotics for UTI in men. The authors found no difference in outcome between trimethoprim, nitrofurantoin and pivmecillinam. However, there was indication of higher treatment failure among nitrofurantoin and pivmecillinam compared to trimethoprim in a subgroup analysis of only *Escherichia coli* UTI cases. This finding is interesting as pivmecillinam and nitrofurantoin are increasingly being recommended and used over trimetoprim for UTI in men in Scandinavia [1,2] due to increased prevalence of resistance to the latter [3,4]. But recent data do not support this to necessarily provide the physician with better treatment alternatives for the male patient presenting with UTI.

We have recently published two large cohort studies on *E. coli* UTI in patients where the general practitioners chose to have the urine sample analyzed in a clinical microbiological department. In the first study, we investigated the outcome of six different oral antibiotics for UTI caused by *E. coli* with and without ESBL production [6], and found pivmecillinam and nitrofurantoin to have the lowest rate of treatment failure (related to the higher prevalence of resistance to the other antibiotics, including trimethoprim). The study adjusted for resistance, sex and age but did not stratify for any of these three covariates. The second study [7] investigated three different regimens of pivmecillinam for UTI—stratified for age and sex and adjusted for resistance—and found that 5 d were better than 3 d (but no different to 7 d) for all age and gender groups. For men, regardless of age, the difference was clinically important as it was well above the widely regarded >10% in risk difference for minimal clinical important difference.

Pivmecillinam and nitrofurantoin have shown excellent efficacy in treating UTI in women and have been recommended as first-line options for UTI in women for a while [8–10]. Thus, it has seemed reasonable to also consider these two antibiotics as alternatives for UTI in men, especially as the high resistance for fluoroquinolones and trimethoprim [3,4] and the newly increased risk awareness for the former [11] have limited the oral options. However, studies investigating nitrofurantoin and pivmecillinam for UTI in men are lacking, even though they are part of the most current recommendations in Sweden (nitrofurantoin or pivmecillinam) [1] and The Capital Region of Denmark (pivmecillinam or trimetoprim) [2].

With regards to the above, we conducted a *post hoc* analysis of the previously used cohort, using the same methodology as in the two original articles [6,7]: a retrospective cohort study conducted between 1 January 2010 and 30 September 2016 in adult males with community-acquired *E. coli* bacteria initiated on trimethoprim, pivmecillinam or nitrofurantoin with indication for UTI (prescription redeemed ±4 d and before the bacteriological diagnosis); long-term prophylactic therapies were excluded; treatment failure measured as redemption of a new antibiotic for UTI or admission to hospital due to UTI within 30 d. The objective was to investigate the three most commonly prescribed antibiotics in Denmark for UTI in men and compare the risk for treatment failure with each other adjusting for resistance, age and for multiple input comparisons.

Interestingly, the results showed trimethoprim to have significantly lower treatment failure than both pivmecillinam and nitrofurantoin, respectively, both when adjusting for the higher trimetoprim resistance or not (Table 1). (This outcome was similar to the findings by Montelin et al. in their *E. coli* group [5]). The results also indicate that the difference in cure rates could potentially be clinically important, as trimetoprim could very well have above 10% lower treatment failure. Thus, although our study comes with several limitations as a retrospective study of men with unknown UTI diagnoses (for details see discussion sections in [6,7]) the difference in outcomes are so substantial that we currently do not believe pivmecillinam or nitrofurantoin should be recommended over trimethoprim for *E. coli* UTI in men based on the higher prevalence of resistance for the latter antibiotic alone. But as a retrospective study of this design comes with obvious limitations and risks for confounding trimetoprim should similarly not be recommended over pivmecillinam and nitrofurantoin based on this finding alone. Before changes to and any firmer recommendations can be made, pharmacological studies to investigate the best dosage in men for these antibiotics with succeeding clinical prospective studies are needed.

**Table 1.** The age distribution, resistance pattern and outcome of non-fluoroquinolones used for UTI in men in the Capital Region of Denmark between 1 January 2010 and 30 September 2016.

Empirical Antibiotic	Cases	Proportion 18–50 years (%)	Proportion 51–70 years (%)	Proportio*n* > 70 years (%)	Absolute treatment failure (%)	Resistant to the redeemed empirical antibiotic (%)	Percent resistant bacteria in treatment failure group (%)	Hazard ratio 30-day treatment failure^a^ (98.75% CI)	*p* Value	Hazard ratio 30-day treatment failure^b^ (98.75% CI)	*p* Value
Trimethoprim 7 d	98	13	34	51	25	14	9/25 (36.0)	Reference	–	Reference	–
		(13.3)	(34.7)	(52.0)	(25.5)	(14.3)					
Pivmecillinam 5 d	903	219	357	327	376	27	15/376 (4,0)	2.05	<.001	1.92	.001
		(24.3)	(39.5)	(36.2)	(41.6)	(3.0)		(1.25–3.37)		(1.17–3.15)	
Pivmecillinam 7 d	1291	293	490	508	529	47	27/529 (5.1)	1.96	.001	1.84	.003
		(22.7)	(38.0)	(39.4)	(41.0)	(3.6)		(1.20–3.20)		(1.13–3.00)	
Nitrofurantoin 7 d	91	8	30	53	43	3	3/43 (7.0)	2.34	<.001	2.15	.002
		(8.8)	(33.0)	(58.2)	(47.3)	(3.3)		(1.28–4.27)		(1.18–3.91)	

a Adjusted for age and resistance.

b Adjusted for age. Level of significance = 0.017 due to multiple comparison.

As for now, the data are insufficient to make any recommendations on which of these three oral antibiotics are to be currently recommended for *E. coli* UTI in men and more studies are much needed.
